# A systematic review of post-migration acquisition of HIV among migrants from countries with generalised HIV epidemics living in Europe: mplications for effectively managing HIV prevention programmes and policy

**DOI:** 10.1186/s12889-015-1852-9

**Published:** 2015-06-19

**Authors:** Ibidun Fakoya, Débora Álvarez-del Arco, Melvina Woode-Owusu, Susana Monge, Yaiza Rivero-Montesdeoca, Valerie Delpech, Brian Rice, Teymur Noori, Anastasia Pharris, Andrew J. Amato-Gauci, Julia del Amo, Fiona M. Burns

**Affiliations:** Centre for Sexual Health and HIV Research, Research Department of Infection and Population Health, University College London, Mortimer Market Centre, off Capper Street, London, WC1E 6JB UK; National Centre of Epidemiology, Instituto de Salud Carlos III, Madrid, Spain; Department of Health and Socio-medical Sciences, University of Alcalá, Alcalá de Henares, Madrid Spain; Ciber de Epidemiologia y Salud Publica (CIBERESP), Barcelona, Spain; HIV & STI Department, Health Protection, Public Health England, England, UK; European Centre for Disease Prevention and Control, Solna, Sweden; Royal Free London NHS Foundation Trust, Pond Street, London, NW3 2QG UK

**Keywords:** Migrants, Sexually transmitted diseases, Surveillance, Epidemiology, HIV prevention, Europe, Prevention & control, Migrant MSM, Sexual behaviour

## Abstract

**Background:**

Migrant populations from countries with generalised HIV epidemics make up a significant proportion of all HIV/AIDS cases in many European Union and European Economic Area (EU/EEA) countries, with heterosexual transmission the predominant mode of HIV acquisition. While most of these infections are diagnosed for the first time in Europe, acquisition is believed to have predominantly occurred in the home country. A proportion of HIV transmission is believed to be occurring post-migration, and many countries may underestimate the degree to which this is occurring. Our objectives were to review the literature estimating the proportion of migrants believed to have acquired their HIV post-migration and examine which EU member states are able to provide estimates of probable country of HIV acquisition through current surveillance systems.

**Methods:**

A systematic review was undertaken to gather evidence of sexual transmission of HIV within Europe among populations from countries with a generalised epidemic. In addition, national surveillance focal points from 30 EU/EEA Member States were asked to complete a questionnaire about surveillance methods and monitoring of the likely place of HIV acquisition among migrants.

**Results & discussion:**

Twenty-seven papers from seven countries were included in the review and 24 countries responded to the survey. Estimates of HIV acquisition post-migration ranged from as low as 2 % among sub Saharan Africans in Switzerland, to 62 % among black Caribbean men who have sex with men (MSM) in the UK. Surveillance methods for monitoring post-migration acquisition varied across the region; a range of methods are used to estimate country or region of HIV acquisition, including behavioural and clinical markers. There is little published evidence addressing this issue, although Member States highlight the importance of migrant populations in their epidemics.

**Conclusions:**

There is post-migration HIV acquisition among migrants in European countries but this is difficult to quantify accurately with current data. Migrant MSM appear at particular risk of HIV acquisition post-migration. Countries that identify migrants as an important part of their HIV epidemic should focus on using an objective method for assigning probable country of HIV acquisition. Robust methods to measure HIV incidence should be considered in order to inform national prevention programming and resource allocation.

## Background

A large proportion of people living with HIV/AIDS in most European Union/European Economic Area (EU/EEA) countries are migrants, that is, people currently living outside their country of birth [1]. In 2013, around one third (33 %; 3 160) of those in EU/EEA who were reported to have contracted HIV through heterosexual contact were migrants from countries with generalised HIV epidemics (mainly sub Saharan Africa) [2]. While most of these diagnoses were made for the first time in Europe, HIV acquisition is predominantly assumed to have occurred in the home country [3].

This assumption is often based on reports from clinicians who make deductive inferences according to a patient’s country of birth, time of arrival in the new country of residence, CD4 cell counts and the natural history of HIV infection. Clinicians may also use algorithms that are biased towards determining that patients born in countries with a generalised epidemic contracted HIV prior to migrating to Europe [4]. Often these estimates do not take into account additional factors. For example, it has been reported that CD4 cell counts close to seroconversion are considerably lower among those living with non-B HIV-1 viral subtypes, which are most common among migrants from outside Europe, particularly in Sub-Saharan Africa. [3, 5, 6]. Consequently, a lower CD4 cell value at diagnosis in those with non-B sub-types may wrongly suggest that individuals have been living with HIV for longer than they have; leading to the conclusion that HIV was acquired before migration. The presence of non-B subtypes among Europe-born populations may also be interpreted as evidence of sexual mixing between migrants and non-migrants.

Funding for primary prevention among migrant communities may be reduced or redirected if surveillance data suggest that individuals do not have HIV prevention needs after they have left their home country or that significant numbers of migrants are coming to Europe as “HIV health tourists” [7, 8]. There have been no previous efforts to collate the information about the proportion of people from countries with a generalised epidemic that contract HIV post-migration. If programme managers and policy-makers underestimate the degree of HIV acquisition within the EU/EEA for migrants from countries with generalised epidemics this may undermine the potential for reducing HIV prevalence and incidence through targeted primary and secondary prevention programmes and policy.

This paper presents a review of the evidence of post-migration HIV acquisition among migrants from countries with a generalised epidemic living in Europe. We examine quantitative studies and surveillance reports based on data on populations from countries with a generalised epidemic which include outcomes that estimate the probable country of acquisition, incident infections, or evidence of sexual mixing. In addition this paper profiles the ability of EU Member States surveillance systems to provide accurate monitoring information about probable country of HIV acquisition. We discuss the implications of these results in HIV prevention programming and policy across the EU/EEA region.

## Methods

### Systematic review

Nine electronic databases (Allied and Complementary Medicine; Cochrane Database of Systematic Reviews; Cumulative Index to Nursing & Allied Health Literature; Database of Abstracts of Reviews of Effects; EMBASE; Health Management Information Consortium; Health Technology Assessment; Medline; PsychInfo) were searched during May 2012^1^. A detailed search strategy was used which combined synonyms for “HIV”, “migrant”, “assortative sexual mixing”, “sexual transmission” with demonyms for all countries with a generalised HIV epidemic (for the complete list search terms, a full description the review protocol please see report [9]). Searches were limited to studies conducted between 01/0/1/2002 and 31/12/2014 to provide the most up-to-date estimates. Studies written in English, French, Italian, Portuguese and Spanish were included. Additional grey literature was retrieved from four websites (United Nations Department of Economic and Social Affairs Population Division; European Health for All database, World Health Organization Regional Office for Europe; European Centre for Disease Prevention and Control), and relevant data were requested from individuals participating in the Member States Survey (see below). The search process was documented by compiling the search strategies used to explore each resource.

#### Selection criteria

Only studies conducted in countries with programmatic or surveillance links within the European Centre for Disease Prevention and Control (ECDC) were included in this review^2^. Studies were eligible for inclusion if: the study population included migrant men or women from countries with a generalised HIV-1 epidemic AND the study included sub-group analysis based on race/ethnicity or country/region of origin OR at least 80 % of the study populations were from countries with a generalised HIV epidemic. Studies were only included if they reported on any of the following outcomes: proportion of target population infected with HIV in country of origin; proportion of target population infected in country of migration; estimate of incident HIV infections (not diagnoses) in target population in country of migration; probable country of infection/HIV acquisition and evidence of sexual mixing. Studies that reported mode of transmission but made no reference to whether sexual transmission took place pre- or post-migration were excluded at full paper screening stage. Qualitative studies (using in-depth interviews, focus group discussions, and document analysis), conference communications, pilots or feasibility studies were excluded.

#### Quality assessment

Studies were selected using a two-stage screening approach. Reviewers devised a checklist to independently screen all retrieved titles and abstracts. Studies were given an overall quality score which incorporated a number of factors drawn from the PRISMA [10] and NICE guidelines [11] including risk of bias, internal and external validity (See Table 1). Papers were graded as having an overall quality score of “Low”, “Medium” or “High”. We were aware that within this review few cohort or intervention studies would be retrieved which may lead to a systemic bias in quality assessment. As a result studies were rated within the paradigm of their study type and studies based on surveillance or cross sectional data were able to achieve overall quality scores of “Medium” or “High”. Studies that received a “Low” score or for which no information to perform quality assessment was available were excluded from the final review. Inter-reviewer reliability scores (Cohen κ) were calculated using Kappa in Microsoft Excel: a kappa of 0.68 for full paper screening and 0.64 for quality appraisal, indicated a high level of agreement between reviewers.

**Table 1:** Criteria used to assess the quality of papers included in full paper review

*Criterion 1: Research question*
Paper is based on a clearly defined research question, which is clearly discussed and referenced throughout the paper.
*Criterion 2: Internal Validity*
The study design was appropriate for the research question and stated study objectives. Selection bias has been minimised; confounding factors have been identified and/or controlled; explanatory variables are based on sound scientific principles; outcome measures are complete and reliable.
*Criterion 3 Clarity of Results*
Results well described and clear appropriate analytical methods used. The precision of association is given or calculable and is meaningful.
*Criterion 4: External Validity*
Source population is well described and the eligible population represent the source population. Selected participants represent eligible population and the results are consistent with results from other studies. The study results are generalisable to the source population.
*Criterion 5: Strength of Association and Statistical Significance*
The study sufficiently powered and precise outcomes have been measured. There are narrow confidence intervals and/or low p-values

#### Data extraction and analysis

Data were extracted using forms detailing study objectives; thematic areas; data collection; methodology (design; setting; population; sample size; geographical scope); results and outcomes (n or %); author defined strengths and limitations and gender specific issues. After data extraction for each paper, studies were grouped according to outcomes of interest. Narrative summaries of each outcome of interest are presented.

### Member states survey

During August 2012, a survey was conducted among 30 EU/EEA Member states using an online survey software package SelectSurveyNet (ClassApps). The questionnaire was developed to gather information from representatives of each member state regarding their knowledge and surveillance of HIV and HIV transmission among migrants from countries with a generalised HIV epidemic. Nationally nominated HIV surveillance contact points were invited to complete the short, 14-item questionnaire which contained mainly open questions, allowing respondents to provide detailed responses. Survey questions were tailored to each country based on information that had been recently submitted to ECDC as part of the Dublin Declaration reporting process^3^. Participants were also able to upload documents to support their responses and these documents were added to the systematic literature review process described above.

## Results

**Fig. 1:**
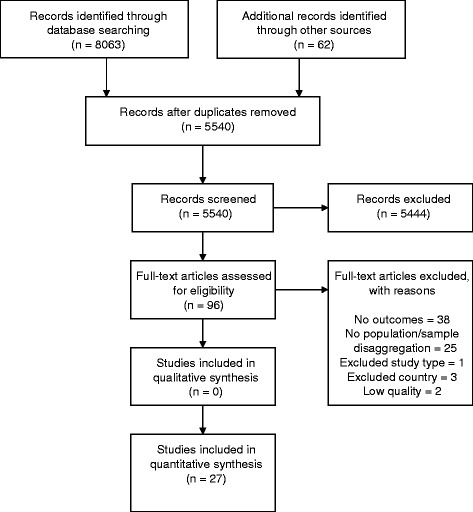
Summary of study selection process

In total 8125 documents were retrieved from all sources. Twenty-seven peer-reviewed papers (representing 26 studies) were found to fulfil the inclusion and quality assessment criteria and were therefore included in the final review. Fig. 1 summarises the outcome of the paper selection process.

Papers were included from six EU countries: United Kingdom (9); Netherlands (4); France (4); Spain (2); Belgium (1) and Italy (3). Two papers were also included from Switzerland and two further papers covered the entire European region. Studies were grouped according to outcomes of interest within three categories: Probable country of HIV acquisition; Estimates of incident HIV infections; and Evidence of sexual mixing. Tables 2 and 3 summarise the 26 studies’ characteristics and includes the quality appraisal process results.

**Table 2:** Summary of included studies: population characteristics

Study reference	Study type	Country	Population	Methods	Sample size
*Probable country of infection and Estimates of incident infection*					
Aggarwal *et al. *(2006) [32]	CS	UK	Black African, white UK-born, and black Caribbean patients	Review of key epidemiologic data obtained from the medical records or from patient’s physician	344 (total) 154 (black African) 42 (black Caribbean)
Burns *et al.* (2009) [38]	CS	UK	HIV-positive Africans within 12 months of initial HIV diagnosis and aged 18 years or more	Data for all respondents to survey ranked for likelihood of acquisition in the UK or abroad	263
del Amo *et al.* (2011) [31]	Systematic review	Europe	Migrant populations/Ethnic minorities	Literature review of the five main databases of articles in English 2005 -2009	37 articles
Dougan *et al.* (2005) [39]	Surveillance	UK	BME MSM newly diagnosed with HIV in E&W between 1997 and 2002	Review of data from two national HIV/AIDS surveillance systems. Undiagnosed HIV prevalence examined by world region of birth	1040
Dougan *et al.* (2004) [40]	Surveillance	UK	Black Caribbean adults	Analysis of voluntary confidential reports of new diagnoses received from virologists and clinicians	528
Dougan *et al.* (2005) [15]	Surveillance	UK	MSM	Analysis of reports of diagnoses from laboratories (since 1985) and clinicians (since 2000)	6386 MSM (total)
Hamers & Downs (2004) [41]	Surveillance	Europe	People living with diagnosed HIV in 12 European countries	Review of HIV/AIDS surveillance databases maintained by EuroHIV network	542,380 (total) 14,077 (migrants from countries with generalised epidemic)
Lot *et al. *(2004) [14]	Surveillance	France	Newly diagnosed HIV positive men and women	Analysis of HIV reports confirmed by laboratories; supplemented by epidemiological and clinical data	1301
Pezzoli *et al. *(2009) [12]	CS	Italy	All adult migrants from a non–EU country registered at primary healthcare centres	Structured questionnaire; included HIV testing; conducted in three cities: Brescia, Rome, Palermo	3003 (total) 674 (sub Saharan Africans)
Rice *et al.* (2012) [18]	Surveillance	UK	Heterosexual adults born abroad and diagnosed with HIV in the UK	National surveillance data analysed; year of infection assigned based on mathematical model	10,612 (total) 9065 (black African)
Rice *et al. *(2014) [19]	Surveillance	UK	Newly diagnosed heterosexual adults seen in care in the UK	National surveillance data analysed for trend changes in probable country of infection.	37,984 (total) 22,524 (black African born abroad)
Semaille *et al. *(2008) [13]	Surveillance	France	Newly diagnosed HIV positive men and women	Analysis of all mandatory anonymous HIV case reports mid-2003 -2006	10,855 (total) 2,511 (confirmed recent infections)
Sinka K *et al. *(2003) [4]	Surveillance	UK	People diagnosed with HIV	Analysis of voluntary confidential reports of new diagnoses received from virologists and clinicians	48,226 (total) 8960 (probably acquired in Africa)
Staehelin *et al. *(2004) [42]	Retrospective cohort	Switzerland	All migrant patients 1984-2000	Single centre retrospective cohort: Time of HIV-infection estimated using CD4 cell count, CD4 cell decline over time and plasma RNA level	1215 (total)
Valin, *et al. *(2004) [43]	CS	France	Sub Saharan Africans, aged 18+ presenting with HIV at outpatient or inpatient appointments	Structured questionnaire collecting socio-demographic & clinical data	250
von Wyl V *et al. *(2011)* [20]	Prospective cohort	Switzerland	Patients who received their HIV diagnosis between 1 January 1996 and 31 December 2009	Phylogenetic analysis – additional demographic data from the Swiss HIV Cohort Study database	1143 individual infected with HIV-1 subtypes A, C,CRF AE, AG
Xiridou *et al. *(2010) [16]	Mathematical model	Netherlands	African migrants, Caribbean migrants, and ‘general’ Dutch population	Model parameterized using data from several surveys including two national surveys	N/A
Xiridou *et al.* (2011) [17]	Mathematical model	Netherlands	African migrants, Caribbean migrants, and the remaining ‘general’ Dutch population	Model describes transmission of HIV in heterosexual partnerships	N/A
***Evidence of sexual mixing***					
Elford *et al.* (2007) [26]	CS	UK	Patients diagnosed HIV infection aged 18+ years	Self-administered questionnaire to patients in six east London public hospitals	1687 (total) 704 (black African heterosexual) 112 (non-white MSM)
Holguin *et al.* (2007) [21]	CS	Spain	Individuals newly diagnosed with HIV-1 infection between 1998 and 2004	Chart review and data analysis	429 (total) 87 (foreign born)
Kramer (2008) [29]	CS	Netherlands	Surinamese and Antillean migrants (incl. 2nd gen) aged 16–70 years	Structured questionnaire administered in social venues	1938
Lai (2013) [24]	CS	Italy	HIV positive patients attending clinical centres	Phylogenetic analysis	254 (total) 114 (Italy) 60 (Africa) 12 (South America) 4 (South East Asia) 64 (other/unknown)
Marsicano *et al.* (2013) [28]	CS	France	Sub Saharan Africans, aged 18–49 living in the Ile-de-France	Interviewer administered face-to-face questionnaire	1874 (total) 973 (women) 901 (men)
Rivas (2013) [25]	Prevalence study	Spain	Migrants from Equatorial Guinea (EG) aged >16 seen for first consultation in in clinic 2002-2008	Analysis HIV & viral hepatitis prevalence among EG migrants compared to migrants from SSA	1493 (total) 1220 (Equatorial Guinea) 276 (other SSA countries)
Snoeck *et al.* (2002) [22]	CS	Belgium	Patients with diagnosed HIV	Phylogenetic analysis & retrospective review of patient records	41 (total, 18 % African)
Tramuto*et al* (2013) [23]	Surveillance	Italy	ART Naïve HIV positive patients attending care	Laboratory hospital surveillance data analysed	155 (total) 113 (native Sicilian) 42 (migrants)
van Veen *et al.* (2009) [27]	CS	Netherlands	Migrants from Surinam, Antilles, Cape Verde & Ghana, aged 18-55	Individuals recruited in community setting so self-complete structured questionnaire	1680

CS = Cross Sectional Study; MSM = Men who have sex with men; BME = Black and Minority Ethnic *Also includes data on Sexual Mixing, E&W = England and Wales

**Table 3:** Outcomes and limitations of included studies

Study reference	Results/Outcomes	Quality scores	Limitations
Aggarwal *et al. *(2006) [32]	*Distribution of non-B subtypes:* Black African 149/154; Black Caribbeans13/42 • *Country of infection B subtypes:* Black African 3 UK (n = 5); Black Caribbean 13 UK, 5 Caribbean, 11 undetermined (n = 29) • *Country of infection non-B subtypes:* Black African 98 before migration, 14 UK, 34 undetermined (n = 149); Black Caribbean: 13/13 UK •*Overall infected in the UK:* Black African 17/154 Black Caribbean: 26/42.	SD: +++ V: +++ G: +++ OS: +++	Poor test specificity differentiating between subtypes B and D likely to be a significant factor in limiting the use of serotyping among black Africans. No standardized prospective data collection. Designation of likely country of infection based, on poorly documented variables from medical records (possible reporting bias). Findings may represent an underestimate of number of infections acquired through overseas travel.
Burns *et al.* (2009) [38]	*Country of Acquisition*: 61(23.2 %) “Definitely acquired HIV abroad”; 44 (16.7 %) “Probably abroad”; 16 (6.1 %), “Definitely acquired in the UK”; 142 (54.4 %) ‘Indeterminate cases’. *All cases (determinate and indeterminate)*: UK acquired: 25.1 % - 35.4 %, Acquired abroad 60.8 % - 67.3 %.	SD: +++ V: +++ G: +++ OS: +++	Acquisition of HIV in UK negatively associated with late presentation therefore findings may underestimate infection acquired in UK. Sample only includes Africans living in London, therefore may not be representative to all HIV positive Africans in the UK.
del Amo *et al.* (2011) [33]	Most studies among sub-Saharan African migrants report infections acquired in the country of origin; includes studies in Denmark, Spain, UK & Canada. Some evidence of post-migration HIV acquisition in EU countries (includes Latin American MSM & other migrant populations); evidence of acquisition during return visits to country of origin.	SD: +++ V: +++ G: +++ OS: +++	The search strategy includes only articles in English; research in other languages, the grey literature and conference abstracts not included.
Dougan *et al.* (2005) [39]	New diagnosis: probable country of infection reported for 38 % of BME MSM. *Born outside and infected in the UK:* 38 % of black African (BA), 27 % of black Caribbean (BC) • *Born and infected outside the UK:* 50 % of BA, 37 % of BC • *Undiagnosed prevalence % (CI): A*ll BME 4.3 (4.1-4.5), BC 4.6 (3.3-6.2), BC 15.8 (11.7-20.8).	SD: +++ V: +++ G: +++ OS: ++	Incomplete data in some variables (country of birth and infection in the new diagnosis study). Heterogeneous population compared for each of the outcomes.
Dougan *et al.* (2004) [40]	*Heterosexual men infected in UK*: 37 (country of birth unknown: 24) • *Heterosexual women infected in UK* 66 (country of birth unknown: 31) • *MSM infected in the UK* 48 (Country of birth unknown: 24).	SD: +++ V: +++ G: +++ OS: +++	Heterosexual transmission of HIV among Caribbeans within UK likely to be underestimated. If exposure to HIV has occurred in more than one country, the country with the highest prevalence will be assigned the likely country of infection. Missing data about country of birth may have had an impact on the review.
Dougan *et al.* (2005) [15]	*Probable country of infection MSM born in Africa*: Infected in Africa =46.4 %; UK = 45.5 %; Other = 8.2 % • MSM born in Caribbean: Infected in Caribbean = 50.0 %; UK = 42.6 %; Other: 7.4 % • MSM born in Asia: Infected in Asia = 30.6 %; UK = 61.2 %;Other = 8.2 %.	SD: +++ V: ++ G: +++ OS: ++	Country of Birth unknown for almost 50 % of sample; results may underestimate the number of diagnosis among MSM born abroad & proportion of MSM infected abroad because of clinician and patient reporting bias. Unclear whether permanent migrants or visitors.
Hamers & Downs (2004) [41]	Most HIV infections diagnosed in migrants probably acquired in country of origin. UK: 75 % of heterosexual infections diagnosed in 2002 probably acquired in Africa • Germany: new diagnoses increased in 2002 among heterosexuals from countries with generalised epidemics, majority infected in their countries of origin • Sweden: > 80 % of HIV infections acquired through heterosexual contact were probably acquired abroad • Denmark: 37 % of all diagnoses in 2002 were among migrants; 59 % infected through heterosexual contact, most infected abroad. • Belgium: 4016/5515 of infections ever diagnosed in heterosexuals were in non-Belgians—mostly Africans.	SD: +++ V: ++ G: +++ OS: +++	Based on secondary data. No clear that reviewed literature was quality assessed.
Lot *et al.* (2004) [14]	*Patients infected through heterosexual transmission:* 690 patients (47 % from SSA). No data on the nationality or ethnicity of MSM • *Proportion of recent infections among heterosexuals:* SSA 26 % vs France 44 % (p = 0.0001).	SD: +++ V: +++ G: +++ OS: +++	Based on preliminary data. Late reporting and longer follow-up periods could show larger differences in recent infections. Additionally, the authors do not report on the ethnicity of patients just country of origin. No data on the nationality of MSM or IDUs.
Pezzoli *et al.* (2009) [12]	HIV-1 detected in 0.97 of participants (95 % CI 0.90–1.2) • *Avidity Testing (n = 27)* Six (22.2 %) probably acquired in Italy by migrants from: SSA (n = 3), eastern Europe (n = 2), and Latin America (n = 1). All 4 (14.8 %) who acquired infection before migration were migrant SSA.	SD: +++ V: +++ G: +++ OS: ++	Recruitment was not evenly balanced between centres; the study acceptance rate was 73.6 %. Place of infection could not be determined for 17 (63.0 %) of 27 persons (this is presumably due to recall bias). Very small sample size for avidity testing.
Rice *et al.* (2012) [18]	*Probable place of infection:* 33 % (26 %-39 %) acquired HIV while living in the UK • *Percentage increased from 24 % (16 %-39 %) in 2004 to 46 % (31 %-50 %) in 2010 (p < 0.01).*	SD: +++ V: +++ G: +++ OS: +++	CD4 cell method may over estimate UK as place of infection since the longer a person is in the UK, the more likely they are to have been assigned UK as place of infection, despite travel habits and behaviour. Missing data for approximately 40 % of eligible adults. Unclear robustness of model used to calculate date of infection.
Rice *et al.* (2014) [19]	*Probable country of infection: The percentage of BA heterosexual adults probably acquiring HIV in the UK increased from 9.1 % (276/3019) in 2002 to 37 % (444/1202) in 2011 (P < 0.01).*	SD: +++ V: +++ G: +++ OS: +++	Definition of heterosexual is based on probable route of infection as reported by clinics, and there is potential for misclassification. (See above for limitations in assigning country of infection).
Semaille *et al.* (2008) [13]	*Proportion recent infections:* French heterosexuals 27 %, SSA heterosexuals 8.4 %. OR for French (Ref.SSA): 3.95 (3.36-4.64).	SD: +++ V: +++ G: +++ OS: ++	Difficult of interpret available data.
Sinka K *et al.* (2003) [4]	*Probable Country of Infection for black African and black other* (n = 7741)*:* UK or Rest of Europe 523/ (6.76 %); Africa: 6163 (79.6 %).	SD: +++ V: ++ G: +++ OS: ++	Limitations of surveillance data due to missing variables, particularly country of birth, ethnicity and country of acquisition. Heterosexual transmission underestimated due to how this data is recorded.
Staehelin *et al.* (2004) [42]	*Infection pre- migration (“with great certainty “or “presumably”)* SSA: 78 (86.5 %) SEA: 13 (50.5 %) • *Infection post-migration* SSA: 2 (2.2 %) SEA: 6 (25 %).	SD: +++ V: +++ G: +++ OS: +++	Source of infection not analysed because of poor availability of data. Sample size of SSA: only 92 patients. The robustness of the methodology for “Time of HIV-infection and migration” was not discussed directly; authors cite evidence there is no difference in the natural history of HIV infection in patients of differing ethnicity.
Valin, *et al.* (2004) [43]	*Probable country of infection:* 44 % SSA, 29 % France, 27 % unknown country.	SD: ++ V: ++ G: +++ OS: ++	Proportion of patients who arrived in France after 1999 (34 %) overestimated. Study population includes naturalized citizens; is not representative of the entire the HIV-positive population originating from sub-Saharan Africa and living in France. Some questionnaire items may be subject to reporting bias.
von Wyl V *et al.* (2011)* [20]	*Proportion of non-B subtype viruses:* Increased from 22 % in 1996 to 33 % in 2009 • Over 80 % of all non-B infections among Africans may have originated outside of Switzerland: 20 % of all sequences from this group were contained within Swiss-specific clusters.	SD: +++ V: +++ G: +++ OS: +++	Sampling bias (substantially alleviated by the high representativeness of the SHCS), linkage between individuals can never be established with absolute certainty.
Xiridou *et al.* (2010) [16]	*“New” Infections (Estimated 1.50 new infections/100,000 people/year):* 53 % of new infections among migrant Africans (32 % acquired in The Netherlands), 26 % among Caribbean Migrants (18 % acquired in the Netherlands).	SD: +++ V: +++ G: +++ OS:++	Data used in model taken from different studies, therefore difficulty to assess research quality.
Xiridou *et al.* (2011) [17]	*Incidence of HIV among heterosexuals:*1.50 new infections per 100,000 individuals per year in 2010 (infections occurring as a result of sexual contacts in The Netherlands or during trips of migrants to their home country).• *Sub-group analysis* 67.18 new infections/100,000 African migrants, 12.12 /100,000 Caribbean migrants, 0.47/100,000 Dutch local.	SD: +++ V: +++ G:N/A OS: ++	Model does not take into account differences between 1st and 2nd generation migrants.
Elford *et al.* (2007) [26]	*Assortative Mixing:* 80 % of BA heterosexual men and women reported sexual partners were also BA.	SD: +++ V: +++ G: +++ OS: +++	High-risk sexual behaviours may be underreported because of social desirability bias or because of the associated stigma. Selection bias from response rate; not broadly representative of those living with HIV as sample exclusively from London.
Holguin *et al.* (2007) [21]	*Prevalence of HIV-1 non-B subtypes and recombinants* 40 (28.8 %) samples, Migrants: 28 (53 % of all migrants in study - 75 % acquired their infection through sexual contact with people born in African) Native Spaniards: 12 (13.7 % of all native Spaniards in the study - 4 most likely acquired HIV-1 through unprotected sex in sub Saharan Africa; 3 with Africans residing in Spain; 2 with partners from Spain; 2 sexual contact with sex workers and 1 MSM with multiple partners).	SD: + V: ++ G: + OS: ++	Number of non-B subtypes among newly diagnosed native individuals is biased and could be underestimated. Subtyping of a large number of samples would be required to determine if the incidence of HIV- 1 non-B variants is increasing over time in the newly diagnosed native population.
Kramer (2008) [29]	*Sexual mixing (sexual partner with differing ethnicity)* High risk = 42 % (84 % unprotected), Moderate risk = 59 % (no data), Low risk = 66 % (no data).	SD: +++ V: ++ G: ++ OS: ++	Convenience sample and social desirability bias Includes both first generation and second generation migrant with no distinction drawn between them in analysis.
Lai (2013) [24]	Sexual mixing: 50 % of men and 47 % of women reported partners born in different countries. Most partners from a different African country (men 19 %; women 20 %).	SD: + V: ++ G: + OS: ++	Convenience sample; low response rate (14 %); desirability bias; data does not support some conclusions reached in the discussion.
Marsicano *et al.* (2013) [28]	*Factors associated with epidemiological networks: Country of origin independently associated with the probability of isolates being detected in clusters OR for Italian vs. African origin: 5.3, 95 % CI: 2.2–12.9, P < 0.001; South American vs. African origin: 25.6, 95 % CI: 2.0–162.0, P < 0.001.*	SD: +++ V: ++ G: ++ OS: +++	ARCA database has relative lack of country of origin and risk factor information for some patients which could have weakened the strength of the detected associations. Clusters were probably underestimated and incomplete due to missing data.
Rivas (2013) [25]	*Proportion of B subtypes*: Total 4 (3.3 %); Migrants from Equatorial Guinea 2(2.9 %); sub Saharan Africa; 1 (5.6 %) P = 0.47.	SD: ++ V:++ G:++ OS:++	Sample disproportionately represented by women and elderly people so might not reflect wider Equatorial Guinea community. Poor justification for some conclusions e.g. low CD4 cell counts = imported infections.
Snoeck *et al.* (2002) [22]	*Country of Infection:* 45 % Africa, 2 % South-America, 6 % rest of Europe or USA. • *Origin of the virus (P = 0.0004):* Belgium (19) Subtype B = 16; Non-B = 3; Other (22) Subtype B = 3; Non-B = 19 • No association between nationality and subtype (P = 0.06).	SD: +++ V: ++ G: ++ OS: ++	Small sample size. Disproportionate numbers of female non-Belgians than male non-Belgians in the study population may have introduced a bias.
Tramuto*et al* (2013) [23]	*Proportion of non-B subtypes:, 107 (69.0 %) were infected with B strains, whereas non-B subtypes were detected in 48 subjects (31.0 %).Only 9.7 % (n = 11/113) of Italian-born subjects were infected with non-B HIV-1 variants. 3 (7.9 %) Africans were infected with B subtypes.*	SD: +++ V: +++ G: ++ OS: +++	Data does not support some of the conclusions. Authors do not acknowledge limitations of surveillance data.
van Veen *et al.* (2009) [27]	*Sexual mixing:* Partners from the same ethnicity 59 %, Partners with differing ethnicity 41 % (15 % with Dutch partners; 21 % with partners of “Other” ethnicity; 5 % with both Dutch and “Other”).	SD: +++ V: +++ G: ++ OS: +++	Desirability bias**;** convenience sample; auto-selection bias.

BA = Black African, BC = Black Caribbean, SSA = Sub Saharan African, SEP = Socioeconomic Position, MSM = Men who have sex with men SD = Study Design, V = Validity, G = Generalisability, OS = Overall Score, N/A = Not applicable

### Probable country of HIV acquisition and estimates of incident HIV infection

Estimates of probable country of infection and/or estimates of incident infection were found in 18 of the 27 papers selected for systematic review. In most of the papers, the study population included people from countries not considered to have a generalised HIV epidemic, but data were disaggregated which allowed reviewers to perform data extraction and compare data across countries. The estimates varied both within countries and across Europe, and covered a range of subgroups, including men who have sex with men (MSM) as well as heterosexuals. Table 4 shows the proportion of infections acquired among sub Saharan Africans post–migration in France, Netherlands, Switzerland and the UK. Table 5 shows infections acquired post-migration among individuals from the Caribbean and Asia in Italy, Netherlands, Switzerland and the UK.

**Table 4:** Proportion of infections acquired in European countries among people born in Africa or with Black African ethnicity

*Author and year*	*Country/City/Region*	*Profile*	*Proportion*
Aggarwal I (2006) [32]	London (UK)	Black African	11
Burns FM (2009) [38]	London (UK)	African	25 – 35
Dougan S (2005) [15]	England and Wales	MSM black African	39
Dougan S (2005) [39]	England and Wales	MSM born in Africa	46
Rice BD (2012) [18]	England, Wales, Northern Ireland	Black African	29
Rice BD (2014) [19]	England, Wales, Northern Ireland	Black African	37
Sinka K (2003) [4]	United Kingdom	Black African	3
Staehelin C (2004) [42]	Switzerland	Sub-Saharan African	2
Valin, N (2000) [43]	Ile-de-France (France)	Sub-Saharan African	29
Xiridou M (2010) [16]	Netherlands	African migrants	32

**Table 5:** Proportion of infections acquired in European countries among people born in Caribbean or Asia or with Black Caribbean ethnicity

*Author and year*	*Country/City/Region*	*Profile*	*Proportion*
Aggarwal I (2006) [32]	United Kingdom	Black Caribbean	62
Dougan S (2004) [40]	England, Wales, and Northern Ireland	Black Caribbean male heterosexuals	24
Dougan S (2004) [40]	England, Wales, and Northern Ireland	Black Caribbean women	41
Dougan S (2004) [40]	England, Wales, and Northern Ireland	Black Caribbean MSM	62
Dougan S (2005a) [15]	England and Wales	MSM black Caribbean	61
Dougan S (2005b) [39]	England and Wales	MSM born in Caribbean	43
Dougan S (2005b) [39]	England and Wales	Asia-born MSM	61
Pezzoli MC (2009) [12]	Italy	Sub Saharan African, Eastern Europe and Latin America	22
Rice B D (2012) [18]	England, Wales, Northern Ireland	Black Caribbean	59
Staehelin C (2004) [42]	Switzerland	Southeast Asian	25
Xiridou M (2010) [16]	The Netherlands	Caribbean migrant	18

While the generilisability and validity of all these papers were medium or high, there are some limitations to the data presented (Table 3). Data estimating the proportion of post-migration HIV acquisition from cross sectional studies in France, Italy, Switzerland and the UK relied on samples that were either very small [12] or were reported to be possibly biased and unrepresentative of the wider population from which the samples were drawn [13–15]. Estimates from The Netherlands were derived from mathematical models. The authors provided very little information about the data used to source the models nor did they discuss the consequent limitations such data placed on the models [16, 17].

Using a new method to ascertain likely country of HIV infection, the UK recently published estimates of place of HIV acquisition among 10,612 heterosexual migrants (9065 of black-African ethnicity) diagnosed with HIV in the UK between 2004 and 2010 [18]. The authors used CD4 at diagnosis to estimate year of infection for each of the adults in their study population and taking into account the variable “Year of Migration”, assigned probable place of HIV acquisition. The CD4-cell based method estimated that 33 % of the study population (26 %-39 %) acquired HIV while living in the UK, three times higher than national estimates of HIV based on clinic reports (11 %). The study also found that the proportion of persons who had acquired their HIV while living in the UK had increased from 24 % (16-39 %) in 2004 to 46 % (31 %-50 % in 2010 (p < 0.01). Similar analysis performed at a later point by the same authors estimated the increase among black African heterosexuals to be from 9.1 % in 2002 to 37 % in 2011 (p < 0.01), however this analysis included data based on clinical reports [19].

The authors discuss two major limitations with this method. First, the method cannot take into account travel (including vacations back to the country of origin) post-migration. Second, the method relies on key variables (CD4 counts, date of arrival and country of birth) and therefore it cannot be applied if these data are missing.

### Evidence of sexual mixing

Fourteen studies examined sexual activity of people after they migrated to Europe (Table 2). Most studies used molecular epidemiology to describe the distribution of non-B subtypes and HIV transmission networks, and in so doing provide estimates of sexual mixing. These studies show that non-B subtypes are prevalent among people born within and outside Europe: in Switzerland, the proportion of non-B subtype virus increased from 22 % in 1996 to 33 % in 2009 [20]; Holguin *et al.* (2007) [21] found that 53 % of migrants and 14 % of native Spaniards in their study in Grand Canary were infected with non-B strains; Snoeck *et al.* (2002) reported a small number of non-B subtypes originated in Belgium (16 %) [22]; in Italy Tramuto *et al.* (2013) estimate that less than one in ten Italian-born individuals in their study were living with non-B HIV-1 subtypes [23]. More detailed analysis from Lai *et al.* (2013) showed that country of origin was independently associated with the probability of patients being detected in epidemiological clusters, although patients from countries with a generalised epidemic were less likely to be detected than those from Italy or South America [24]. Rivas *et al.* (2013) provide some evidence of sexual mixing by reporting a small proportion of B subtypes (3.3 %) among migrants from sub Saharan Africa living in an area of Spain [25].

Elford *et al.* (2007) [26] state that 80 % of black African heterosexuals reported sexual partners of the same ethnicity and van Veen *et al.* (2009) [27] found that 41 % of their sample had partners with different ethnicity (15 % with Dutch partners). Similarly Marisciano *et al.* (2013) reported that 50 % of men and 47 % of women had partners from a different country although most were from another African country [28].

In a cross sectional survey examining sexual behaviour among Surinamese and Antillean migrants in the Netherlands [29], the authors describe travel patterns of migrants and provide estimates of sexual mixing for a population they classified as having the potential to provide a transmission “bridge” between the Caribbean and The Netherlands. Those having unprotected sex in both countries, the so-called “bridge population”, reported partners of a different ethnicity less often than those in moderate or low risk groups (42 %, 59 % and 66 % respectively) [29].

### Member states survey

Twenty-four countries responded to the survey (response rate: 80 %). Five countries reported having data on sexual transmission of HIV among migrant communities. Denmark and Germany provided some contextual information about sexual transmission of HIV, but were unable to provide estimates of the proportion of migrants acquiring their HIV post-migration. The Netherlands provided grey literature and peer reviewed papers (subsequently included in the literature review) showing within-country sexual transmission among migrant communities. The most specific data came from Norway (14 % of 152 migrants diagnosed with HIV in 2011 were thought to have acquired their HIV infection post-migration) and the UK, (see Rice *et al.*[18]).

The remaining member state respondents did not provide data on, or estimates of, sexual transmission of HIV among migrant communities.

#### Ascertaining probable country of infection

Fifteen countries include a *Probable Country of Infection* (PCOI) data field in their new diagnoses database. Survey respondents reported that PCOI is established by direct interview of the case or indirectly through a clinician report. A range of data are used to assign PCOI – in particular a combination of clinical (such as CD4 count and viral load at diagnosis) as well patient demographic information (COB and *Date of Arrival*). Thirteen countries collect enough data to use the CD4-cell based method of calculating PCOI developed by Rice *et al.* (2012) (Belgium, Denmark, France, Greece, Italy, Lithuania, Luxembourg, Malta, Portugal, Romania, Slovak Republic, Sweden, UK).

## Discussion

This study draws together evidence on the extent to which sexual acquisition of HIV is occurring among migrants from countries with a generalised HIV epidemic after they have moved to the EU/EEA. Despite this systematic review of the literature, survey of EU/EEA Member states, and data reported by countries as part of monitoring the Dublin Declaration which all provide some evidence, it remains difficult to gain an accurate picture for the EU/EEA. The published literature is relatively sparse: 27 papers were retrieved from just seven countries. Pan-European studies were also included, but these were low on detail and did not provide specific estimates.

The data do provide evidence of on-going post-migration HIV acquisition, most notably by providing estimates of probable country of acquisition or of incident infection. The methodology for calculating these estimates varied across studies, making it difficult to assess their reliability and comparability. Figures for HIV infections contracted post-migration ranged from as low as 2 % among sub Saharan Africans in Switzerland, to 62 % among black Caribbean MSM in the UK. As this example demonstrates, study populations varied, with some samples based on country of birth, others ethnicity and some using both definitions as surrogate markers for migration. Such heterogeneity in the retrieved data makes it difficult to construct a succinct and clear understanding of on-going sexual acquisition and transmission, both within host countries or across the EU/EEA. Nonetheless, all studies found that post-migration acquisition of HIV is occurring and this must be measured and understood in order to adequately meet the HIV prevention needs of migrant communities.

It is possible that limitations in our review methodology prevented us retrieving relevant information. We were unable to include papers published in languages outside our language skill set. No papers were retrieved from the eastern part of the EU, though this is not unexpected as Eastern Europe does not have a large population of migrants from countries with a generalised HIV epidemic. A survey of EU/EEA Member States combined with data captured through the monitoring of the Dublin Declaration on Partnership to Fight HIV/AIDS in Europe and Central Asia were expedient and cost-effective ways of gathering data about specific countries. Nonetheless, these often relied on the knowledge and experience of one representative from a national body who may not have had all relevant information at their disposal.

### Implications for HIV surveillance

Understanding and quantifying HIV transmission in a given population is complex. Effective practice would be to monitor trends in HIV incidence, but these measures are not straightforward and are often beyond the scope of routine surveillance systems. Trends in new HIV diagnoses are sometimes used as a proxy for incident cases, but these are also subject to testing biases as they are dependent on high uptake of regular HIV testing in the population of interest. New tests of recent or incident infections may provide greater insights into real time transmission dynamics. Another challenge is specific to HIV surveillance among migrant communities. Migration as a process is not static, and many migrants travel backwards and forward between their country of origin and country of residence, making estimates of place of infection subject to measurement error. Studies addressing sexual mixing patterns and sexual activity of migrants from countries with generalised epidemics do not necessarily make the best proxies when attempting to find evidence about probable country of HIV acquisition. For example, the increasing proportion of non-B subtypes shown in these papers may reflect on-going migration rather than post-migration HIV acquisition or transmission.

This study found that many more member states collect data that could be used in an objective method for assigning *Probable Country of Infection* than is expected from the published data. The lack of published evidence could indicate that this is an area requiring technical support to EU/EEA Member States to carry out this work. Additionally, it is possible that some publication bias exists, with authors unable or unwilling to publish data on a sensitive topic that may have a stigmatising impact on migrant communities particularly within the context of Europe’s ongoing immigration debate [7, 30].

EU/EEA Member States might be encouraged to analyse and publish such data if standardised methods of calculating *Probable Country of Infection* were developed and implemented in surveillance systems across the region. To achieve this a minimum dataset of CD4 cell count at diagnosis, date or year of arrival and country of birth on all newly diagnosed persons are likely to be required. In some settings, sentinel surveillance or repeat cross sectional surveys, could play an important role in providing this necessary evidence. Member States might also be encouraged to publish data if they formed meaningful partnerships with migrant and/or minority ethnic community organisations who could provide support and guidance about how to disseminate data sensitively [31]. Data relevant to post-migration sexual acquisition could then be used to plan and monitor HIV programmes that meet the needs of migrant communities in Europe.

### Implications for prevention programming

Given the evidence for post-migration HIV acquisition among migrant groups in some EU/EEA Member States, there is a need for increased awareness among policy-makers of the HIV prevention needs of migrants from countries with generalised epidemics. This awareness will require additional attention and resources to improve primary prevention programmes targeted to the specific (culturally appropriate) needs of various migrant communities.

Many migrants remain sexually active during transit and after they have reached their destination country. Papers examining sexual mixing present evidence that the number and proportion of non-B HIV-1 subtypes are increasing in non-migrants and established minority ethnic communities across Europe [20–22, 32]. The propensity for assortative sexual mixing means that people from countries with generalised epidemics may continue to live in a community with a generalised epidemic even after they have moved to Europe, but be unaware of their HIV prevention needs in their new home.

Migrant MSM appear at particular risk of HIV acquisition post-migration. Behavioural data suggests assortative sexual mixing according to country of origin or ethnicity is not a prevailing feature of sex between men [33] as reflected in the predominance of B subtypes in these communities [32]. Acquisition of viral clades not prevalent in home countries supports post-migration acquisition and (as with heterosexual men) this highlights the need for primary prevention programs targeting these communities. Effective HIV prevention interventions would need to recognise that many MSM from countries with a generalised epidemic may not self-identify as gay men or disclose their sexual identity [34]. Surveillance data does not routinely report on migrant MSM and as such are unable to inform prevention programmes for this population.

### Implications for policy

Countries who identify migrants as an important part of their country’s HIV epidemic should consider developing an evidence-based, long-term policy to introduce prevention programmes that reduce HIV-acquisition in these groups. To reduce acquisition countries would need to include policies around structural, behavioural and biomedical prevention interventions that are targeted to all communities, including migrants from countries with generalised epidemics.

Approximately half of the EU/EEA countries surveyed report that they do not provide ART to irregular/undocumented migrants, that is, to persons that cannot legally reside in the country [35]. Treatment as a means of reducing sexual transmission of HIV now forms a key part of the prevention paradigm, like other conditions of paramount public health importance such as tuberculosis [36]. However, policy responses such as mandatory screening contravene the WHO policy framework for HIV testing in Europe which states mandatory HIV testing for migrants and asylum seekers upon arrival violates basic rights and ethical principles and cannot be justified on public health grounds [37]. Improving access to HIV treatment for all infected persons, regardless of their administrative and or immigration status, could positively impact on reducing incident infections both within and beyond migrant communities. This would necessitate addressing the already identified barriers to HIV prevention, testing and care that exist for migrant communities [31]. Failure to ensure access to HIV treatment for all persons in need could prove detrimental to efforts to ameliorate the HIV epidemic.

## Conclusion

There is limited published evidence about the acquisition of HIV within Europe among migrants from countries with a generalised epidemic. Individuals are certainly contracting HIV through sexual contact after they have moved to Europe and MSM appear to be at particular risk of post-migration HIV acquisition, yet this is rarely acknowledged within the literature. Only a few countries collect and publish data to enable robust estimates to quantify or monitor the place of HIV infection, which may have a detrimental impact on HIV prevention interventions.

The majority of countries that identify migrants as an important at risk population for HIV infection have put in place measures to estimate the distribution of HIV in these groups. Despite the many areas of concordance and agreement in Member States surveillance systems there remain a number of gaps in the processing and availability of this data. This therefore limits the ability of policymakers and programme managers who wish to have an impact on HIV incidence at country and regional levels.

## Footnotes

1 The search was updated on 9th January 2015 to include articles published between January 2012 and December 2014.

2 These are EU/EFTA countries: Austria; Belgium; Bulgaria; Cyprus; Czech Republic; Denmark; Estonia; Finland; France; Germany; Greece; Hungary; Iceland; Republic of Ireland; Italy; Latvia; Liechtenstein; Lithuania; Luxembourg; Malta; The Netherlands; Norway; Poland; Portugal; Romania; Slovakia; Switzerland; Slovenia; Spain; Sweden; UK.

3 European Centre for Disease Prevention and Control. Thematic report: Migrants. Monitoring implementation of the Dublin Declaration on partnership to fight HIV/AIDS in Europe and Central Asia: 2012 Progress Report. Stockholm: ECDC; 2013.
